# Disease and Treatment Perceptions Among Asian Americans Diagnosed with Chronic Hepatitis B Infection

**DOI:** 10.1007/s11606-013-2673-0

**Published:** 2013-12-19

**Authors:** Kalman Tokes, Syed Quadri, Patrick Cahill, Grace Chiu, Angel Ivanov, Hong Tang

**Affiliations:** 1 Bristol-Myers Squibb, 777 Scudders Mill Road, Plainsboro, NJ USA; 2 GC Global Research, Brooklyn, NY USA

**Keywords:** chronic hepatitis B, Asian American, survey, treatment, antiviral

## Abstract

**BACKGROUND:**

In the US, over 1 million Asian Americans are estimated to be living with chronic hepatitis B (CHB). Research has shown low awareness of CHB and different attitudes towards its treatment among the diverse ethnicities of Asian Americans.

**OBJECTIVE:**

This study aimed to understand the perceptions and attitudes of CHB treatment among Asian Americans diagnosed with CHB who were either treatment-naïve or being treated for CHB with oral antivirals, and to understand the relative importance of different clinical and economic attributes of oral antivirals that affect CHB treatment decisions and choices.

**DESIGN:**

Face-to-face structured survey administered to participants at central research facilities by interviewers of each participating ethnicity.

**PARTICIPANTS:**

CHB patients from Chinese, Korean, and Vietnamese communities of New York metropolitan, San Francisco/Bay, and Los Angeles/Orange County areas.

**MAIN MEASURES:**

A ‘conjoint’ exercise (discrete choice model) assessed the relative impact of treatment attributes on treatment choice. Implicit “trade-off” decisions made by respondents were estimated using a hierarchical Bayesian model.

**KEY RESULTS:**

Among 252 participants, 36 % were Chinese, 34 % Vietnamese, and 31 % Korean; 56 % were treatment-naïve and 44 % were being treated with an oral antiviral for CHB. The majority (88 %) believed that, if left untreated, CHB can lead to serious liver damage; 72 % believed there are effective prescription medications to treat CHB; and 39 % showed reluctance to be on long-term therapy for CHB because of concerns over side effects. Long-term risk of kidney damage was given the highest relative importance (38 %) when choosing CHB treatment, followed by medication cost (23.4 %), long-term risk of bone thinning (18 %), long-term efficacy (9 %), time on US market (6.8 %), and number of patients treated globally (4.9 %). Results were consistent across ethnicities.

**CONCLUSIONS:**

Patients need access to improved education regarding CHB disease progression, its management, disease outcomes, and the importance of long-term treatment of the disease.

**Electronic supplementary material:**

The online version of this article (doi:10.1007/s11606-013-2673-0) contains supplementary material, which is available to authorized users.

## INTRODUCTION

Chronic hepatitis B (CHB) remains a significant global health burden, despite widespread hepatitis B virus (HBV) vaccination programs. In the US, estimates suggest that more than 2 million people are living with CHB^1^^,^ 2 and that the clinical sequelae of untreated CHB, such as cirrhosis, hepatic decompensation, and hepatocellular carcinoma (HCC), contribute to up to 4,000 deaths per year.^1^^,^ 2 Effective antiviral therapy can reduce progression to cirrhosis and minimize the risk of HCC;^3^^–^ 5 however, in the US, HBV antiviral treatment is prescribed to fewer than 50,000 people per year.^6^ Possible reasons for the apparently low treatment rate include insufficient screening and diagnosis and a need for better education and referral, particularly for disproportionately infected populations. Approximately half the cases of CHB in the US are among Asian American individuals who have migrated from areas with a high prevalence of HBV infection.^1^^–^ 3 The Asian American population is a rapidly growing and diverse community, estimated to increase from 4.8 % of the US population in 2010 to 9 % in 2050.^7^^,^ 8 Asian American individuals generally acquire HBV infection perinatally or early in life, and have a high risk of progression to CHB and subsequent long-term complications including HCC. Early diagnosis and appropriate, effective treatment are therefore of particular importance in this population, and it is desirable that primary care physicians are aware of what key factors influence the choices that Asian American patients make and barriers that may prevent them from seeking treatment.

Previous research has shown variable awareness of CHB among the diverse ethnicities of the Asian American population and different attitudes towards CHB treatment for Asian American patients among primary care providers.^3^^,^ 9 ^,^ 10 Several studies have indicated that lack of knowledge about HBV transmission and its consequences leads to low levels of vaccination and screening. A number of initiatives have been implemented to raise the uptake of vaccination and screening in the Asian American population, with some success.^11^^–^ 14 To our knowledge, no studies have specifically evaluated the level of awareness among Asian American individuals of current potent antiviral therapies available for the treatment of CHB.

This study aimed to evaluate perceptions and attitudes about CHB treatments among Asian American individuals diagnosed with CHB, and to assess factors affecting treatment decision and choice.

## METHODS

The primary objective of this study was to determine, among Asian Americans diagnosed with CHB (treatment-naïve or already being treated for CHB with oral antivirals), the relative importance of different attributes of oral antivirals in making treatment decisions and product choices. A key secondary objective was to assess general attitudes toward CHB treatment among Asian Americans diagnosed with CHB.

### Participants

During November and December of 2011, participants were recruited from Chinese, Korean and Vietnamese communities, since these represent the Asian American populations with the highest prevalence of CHB.^3^ Sources included GC Global’s panels; grass-root recruitment efforts at local community centers; health centers, doctors’ offices and clinics in the local Chinese, Korean and Vietnamese communities; and networking and referrals from families and friends of CHB patients, non-medical staff, community workers and social workers who work with or know of CHB patients in these communities. Participants were screened to meet key target criteria: 18–65 years old; Chinese, Korean, or Vietnamese ethnicity; with a doctor’s diagnosis of CHB at least 6 months previously; on current antiviral treatment for CHB treatment (Treated) or naïve to CHB antiviral treatment (Treatment-naïve); and with no participation in CHB studies in the last 6 months. Quotas were applied to achieve a good balance in the sample in terms of age, gender, treatment status, ethnicity, and region of the US. Screening included criteria designed to ensure that respondents had at least a minimum level of concern about their CHB condition: must have CHB check-ups with their doctor at least every 1–2 years (or more often); must not be totally opposed to the idea of taking Western medicines to treat CHB (“not receptive” and “not at all receptive” responders were screened out).

### Survey

Study data were collected during December 2011 using a structured online survey, including the following sections: demographic data, treatment status, a ‘Conjoint’ exercise (using a discrete choice model), cost sensitivity, and treatment attitudes. The survey was translated into Chinese, Korean, and Vietnamese, and was administered at central research facilities by trained interviewers of each participating ethnicity who entered participants’ responses into the online database. Participants were given the option of conducting the interview in their native Asian language or in English.

### Conjoint Exercise

To assess what attributes of oral antivirals have the greatest influence on the treatment choices that participants make, a conjoint exercise was carried out. The conjoint exercise used a discrete choice model^15^ that required participants to choose between pairs of hypothetical products characterized by a set of six product attributes: long-term efficacy, 5-year risk of thinning of bones, 5-year risk of kidney disease, weight of evidence (number of patients treated), weight of evidence (number of years in the market) and cost, as detailed in Table 1. The chosen characteristics are derived from previous quantitative and qualitative research studies as being likely to have some relevance as consideration factors for Asian patients (GC Global data on file). For each hypothetical product, each of the attributes could be set at one of three or four possible levels, reflecting the ranges reported in the literature. For each of the attributes, one or two of the possible levels accurately reflected levels reported for one or more of the antiviral therapies available at the time of the survey,^16^^–^ 20 while the other levels were outliers. Such a broad range of levels improved the measurement of trade-offs, since the difference across the range was big enough to make participants’ choices clear. A total of 64 product comparison scenarios (comparisons between two hypothetical products) were used. These scenarios were selected using an orthogonal design and were divided into eight blocks, each with eight different scenarios using a balanced design, so that within each block, all respondents saw all levels of each attribute proportionally (Online Supplementary Figure S1). No two products or scenarios used in the design were exactly alike. In each scenario, participants were asked to choose between the two hypothetical products (coded with letters), with the assumption that both were approved and deemed suitable for them by their physician. Each participant was presented with one block of eight scenarios. Each block was seen by an equal number of Treated and Treatment-naïve participants. Care was taken to ensure that the attributes were described in patient-friendly terminology. Interviewers made sure that each participant fully understood both the terminology and the requirements of the conjoint exercise before starting it.

**Table 1:** Attribute Definitions Used in the Discrete Choice Model and Product Preference Analysis

Attribute	Description	Possible levels (used in the discrete choice model)
Long-term efficacy	Doctors’ estimate of the probability that the medicine will continue to work well for 5 years	71 %
		85 %
		92 %
5-year risk of thinning of bones *[bone mass density]*	Doctors’ estimate of the probability that you will have thinning of bones if you take the medicine for 5 years	< 1 %
		7 %
		14 %
5-year risk of kidney disease *[renal toxicity]*	Doctors’ estimate of the probability that you will have kidney damage if you take the medicine for 5 years	< 1 %
		10 %
		20 %
Weight of evidence *[level of use]*	How many patients have been prescribed the medicine worldwide	100,000
		200,000
		400,000
Weight of evidence *[time in market]*	How many years the medicine has been approved in the US	2 years
		4 years
		6 years
Cost	Out-of-pocket cost of this medicine to you each month, assuming that you will have to take it for at least 12 months	$0
		$50
		$100
		$150

Statistical processing of the implicit “trade-off” decisions made by participants in each of the product comparison scenarios used hierarchical Bayesian methodology. This method enables the use of a limited number of scenarios with limited attributes, and thus avoids potential survey fatigue, which can reduce the reliability of the data. To capture sample variation among the participants, data analysis was conducted using the statistical software “RJAGS 3.2” (www.cran.r-project.org) to estimate individual level parameters using Markov Chain Monte Carlo (MCMC). The simulations use these individual level parameters to compute share at an individual basis, which is added across to get the aggregate share. Two main types of information were generated: the relative importance of attributes, which describes the weight or importance that each attribute has in determining the overall appeal of a product, with the total relative importance of all attributes adding up to 100 %; and the relative preference, providing an indication of the percent of participants who would be likely to choose a particular product, based on its configuration of attribute levels. A sensitivity analysis was conducted to assess the impact on the relative preference for a product caused by changes in the levels of its individual attributes. This analysis was initiated from a “base case” product configuration, with one attribute flexed at a time, while holding all others constant at base case levels.

### Cost Sensitivity Analysis

Following the conjoint exercise, all respondents were shown a product configuration that matched the attribute levels of a hypothetical optimal product, and were told to assume that their physician had recommended that they start taking this medication. They were then asked to state the likelihood that they would take the medication, at seven different out-of-pocket monthly costs ($0, $50, $100, $150, $200, $250, and $500), presented in random order. Likelihood was scored on a 5-point scale: 1 = definitely not, 2 = quite unlikely, 3 = may or may not, 4 = quite likely, 5 = definitely. They were also asked to specify the maximum acceptable monthly cost for this medication. Cost-sensitivity analyses were performed using a linear model, with reported beta estimates.

### Treatment Attitudes

A battery of statements about treatment attitudes was included in the survey to assess the attitudinal profile of the sample and to provide ongoing longitudinal data in this area. Responses scored the statements on a scale of 1 to 5 (1 = completely disagree, 2 = mostly disagree, 3 = somewhat agree, 4 = mostly agree, 5 = completely agree). Differences in responses between Treated and Treatment-naïve patients were assessed for statistical significance using a two-tailed *T*-test (significance level *p* = *0.05*) using the SPSS software.

## RESULTS

### Sample Composition

A total of 252 participants completed the survey. The participant sample provided a balance across demographic subgroups (Table 2). The majority of participants (141, [56 %]) were Treatment-naïve, with 111 (44 %) on current CHB antiviral treatment, mostly with entecavir, tenofovir, or adefovir, reflecting the current US treatment pattern. The median time since diagnosis was 4 years among patients on current antiviral treatment and 5 years among Treatment-naïve patients. Around 60 % of participants had been diagnosed with CHB in the last 5 years.

**Table 2:** Participant Demographics and Treatment Status (*N* = 252)

Classification		*n* (%)	
Gender	Male	128 (51)	
	Female	124 (49)	
Age	18–45	138 (55)	
	46–65	114 (45)	
Region	New York/New Jersey	85 (34)	
	San Francisco/Bay Area	85 (34)	
	Los Angeles/Orange County	82 (32)	
Ethnicity	Chinese	90 (36)	
	Vietnamese	85 (34)	
	Korean	77 (31)	
Place of birth	USA	18 (7)	
	Asia*	234 (93)	
Education	Less than high school	12 (5)	
	High school graduate	58 (23)	
	Some college/university	56 (22)	
	University graduate/Post graduate	126 (50)	
Employment status	Full-time employed	111 (44)	
	Part-time employed	57 (23)	
	Self-employed	20 (8)	
	Not employed/student	40 (16)	
	Homemaker	13 (5)	
	Retired	11 (4)	
Household income	< $20,000 per year	40 (16)	
	≥ $20,000–$35,000 per year	64 (25)	
	> $35,000–$50,000 per year	49 (19)	
	> $50,000–$75,000 per year	46 (18)	
	> $75,000–$100,000 per year	25 (10)	
	> $100,000 per year	16 (6)	
	Prefer not to say	12 (5)	
		Treated (*N* = 111)	Treatment-naïve (*N* = 141)
Current medication	Entecavir	51 (46)	NA
	Tenofovir	28 (25)	
	Adefovir	27 (24)	
	Lamivudine	4 (4)	
	Unknown	1 (1)	
Frequency of CHB doctor visits	Once per month	7 (6)	0
	Every 3 months	45 (41)^†^	13 (9)
	Every 6 months	45 (41)	60 (43)
	Once per year	8 (7)	51 (36)^†^
	Once every 1–2 years	6 (5)	17 (12)^†^
Medical insurance	Total with insurance	87 (78)	108 (77)
	Employer plan	34 (39)	59 (55)^†^
	Family Health Plus^*‡*^	19 (22)^†^	10 (9)
	Other self-paid	8 (9)	16 (15)
	Medicare^§^	9 (10)	12 (11)
	Medicaid^∥^	12 (14)	7 (6)
	Unknown	5 (6)	4 (4)

*NA* not applicable

^*^Mean length of residence in USA 16 years

^†^Significantly higher than the other group (*p* < 0.05)

^‡^Family Health Plus is a US public health insurance program for adults who have income too high to qualify for Medicaid

^§^Medicare is a social insurance program administered by the US government for Americans over 65 years of age and younger persons with disabilities, end-stage renal disease or Lou Gehrig’s disease

^∥^Medicaid is the US means-tested health program for persons with low income

### Conjoint Exercise (Discrete Choice Model)

The relative impact of product attributes on product choice in the discrete choice model for the total group is shown in Table 3. The attributes that had the greatest impact on product choice were the risk of kidney damage (38 %) and out-of-pocket cost (23 %), between them accounting for over 60 % of total relative importance. The risk of bone thinning was also an important factor, accounting for 18 % of total importance. Long-term efficacy, within the range used in this study (71–92 % efficacy), was of lower relative importance (9 % of total). ‘Weight of evidence’ factors had relatively little importance in selection decisions: time in the US market (7 % of total) and number of patients worldwide (5 % of total).

**Table 3:** Relative Impact of Product Attributes on Product Choice in the Discrete Choice Model, Overall and by Ethnicity and Treatment Status

% relative importance of attributes	Total (*N* = 252)	Ethnicity			Treatment status	
		Chinese (*n* = 90)	Korean (*n* = 77)	Viet (*n* = 85)	Treated (*n* = 111)	Naïve (*n* = 141)
Long-term (5-year) risk of kidney damage	37.9	37.8	38.0	38.0	36.6	38.7
Monthly out-of-pocket cost	23.4	23.5	23.4	23.4	27.9	20.9
Long-term (5-year) risk of bone thinning	18.0	18.4	17.6	17.9	13.2	20.7
Long-term (5-year) efficacy	9.0	9.0	9.0	8.9	7.2	10.0
Time approved in US market	6.8	6.5	7.1	6.8	7.5	6.4
Level of use (no. of patients worldwide)	4.9	4.7	4.9	5.0	7.7	3.3

The relative importance of attributes was highly consistent across ethnicities (Table 3). Results were also broadly consistent by treatment status (Table 3), except that Treated participants appeared to be more sensitive to out-of-pocket cost than Treatment-naïve participants. In addition, Treatment-naïve participants ascribed greater importance than Treated participants to the risk of bone thinning and to long-term efficacy.

**Figure 1:**
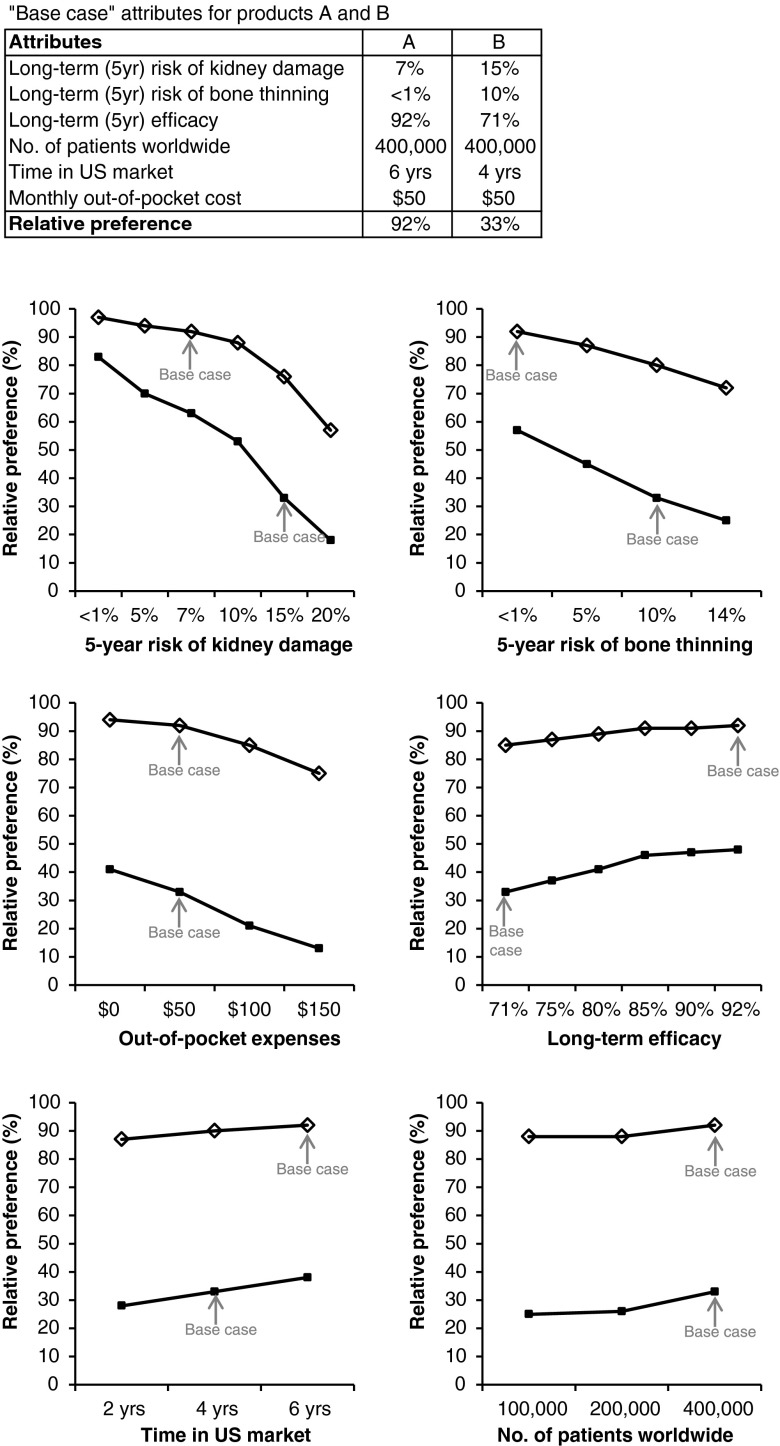
Discrete choice model sensitivity analysis showing the impact on the relative preference for hypothetical products A (◊) or B (▪) caused by changes in the levels of their individual attributes. Base case attributes for each product are shown in the table at the top of the figure. Each figure panel represents the effect of changing the levels of one product attribute at a time, while all other attributes for each product remain at base case (indicated by the *arrows* on each panel).

The results of the sensitivity analysis assessing the impact of changing individual attributes on the preference for two hypothetical products ‘A’ and ‘B’ are shown in Figure 1. Changes to the risk of kidney damage had the highest impact on overall product preference, with a large range in relative preference from the “best case” (< 1 % 5-year risk) to “worst case” (20 % 5-year risk) levels. Relative preference dropped sharply when the long-term risk started to exceed 10 %. Changes in the risk of bone thinning and in out-of-pocket cost both had quite a high impact on relative preference over the range assessed. Progressive increases in the risk of bone thinning reduced relative preference at a fairly rapid rate. The influence of out-of-pocket cost on product preference was more noticeable as the cost exceeded $100/month, suggesting this to be an important psychological barrier. The change in relative preference between “best case” and “worst case” levels was fairly small in the case of long-term efficacy, time in the US market, and number of patients worldwide.

### Cost Sensitivity

**Figure 2:**
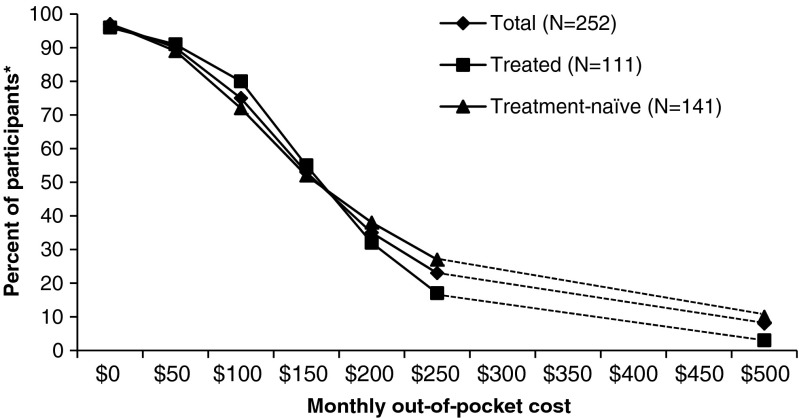
Likelihood of taking Product A at different monthly out-of-pocket costs, among all participants, Treated participants, and Treatment-naïve participants. *Percent of participants responding ‘definitely’ or ‘quite likely’ to the question “How likely would you be to take this medication under each of the following monthly costs (assuming the regimen takes at least 12 months)?” $300, $350, $400, $450 price points were not measured; the dotted lines represent the extrapolated trend.

The results of the cost-sensitivity analysis are shown in Figure 2. Between $100 and $250, a 10 % drop in projected willingness to pay for treatment was seen for each $25 cost increase among Treated patients and for each $35 cost increase for Treatment-naïve patients. Overall, participants indicated an apparent willingness to pay monthly out-of-pocket costs in excess of the actual levels reported by those who were taking CHB treatment at the time of the survey. When shown the seven different cost levels for Product ‘A’, approximately 75 % of participants indicated a willingness to pay $100/month, with around 50 % willing to pay $150/month. When asked to specify a maximum acceptable cost, the mean response among all participants was $138/month (median $100/month). In contrast, around 80 % of Treated patients indicated that they were currently spending ≤ $50 per month in out-of-pocket costs (mean $42/month, median $15/month).

### Treatment Attitudes

**Figure 3:**
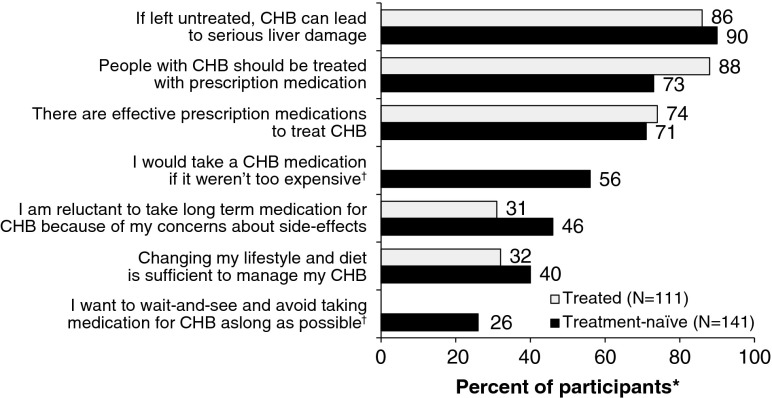
Treatment attitudes by treatment status. *Percent of participants who completely or mostly agree with the statement. ^†^Questions only shown to Treatment-naïve participants.

The majority of patients (88 % overall) acknowledged the seriousness of CHB and support the need for antiviral medication (Fig. 3). Overall, 72 % believed that there are effective prescription medications to treat CHB. A substantial minority (particularly among Treatment-naïve patients) showed reluctance to be on long-term therapy for CHB, because of concerns over side effects. A similar proportion believed that a change in lifestyle and diet would be sufficient to manage their CHB.

## DISCUSSION

The study findings provide important insights about the perceptions and attitudes of CHB medications among Asian Americans already diagnosed with CHB or on treatment for CHB. The assessment of treatment attitudes showed that the great majority of participants (> 70 %), both Treated and Treatment-naïve, acknowledged the seriousness of CHB, supported the need for effective antiviral treatment, and were aware of available treatments. An earlier survey carried out in 2007 among Asian American individuals from Chinese, Korean, and Vietnamese communities, randomly selected from telephone directories, reported a slightly lower awareness of available treatments (around 50 %).^10^ The reduced awareness in the earlier study can be explained by the inclusion of participants without CHB. Increased awareness in our 2011 study may also reflect increased experience with potent antiviral treatments, the impact of updated treatment guidelines (issued in 2009),^5^ and the impact of targeted public health campaigns launched since 2007.^21^ Despite this increased awareness of treatments, a substantial minority of participants (close to 50 % among the Treatment-naïve) was reluctant to take long-term medication because of concerns about potential side effects, and almost as many believed that lifestyle and diet would be sufficient to manage their disease. These results are consistent with the results of the 2007 study, in which the majority of participants expressed concern about possible treatment side effects (61 %) and almost 20 % believed that herbal medicines offered a better treatment alternative.^10^ The high level of belief that diet and lifestyle can be used to manage disease may have a cultural influence. Many Asian cultures place high value on herbal medicines and traditional remedies, and cultural barriers have also been cited as hindering screening in this population.^10^

Concerns about potential side effects were also apparent in the results of the discrete choice model. Attributes pertaining to the long-term risk of side effects were shown to have substantially the greatest impact on product choice. Considering the chronic nature of the disease, concerns over long-term side effects are not surprising. However, there is a large body of evidence supporting the safety and tolerability of long-term antiviral therapy in both global and Asian CHB populations.^22^^–^ 25 It is important that Asian Americans are educated about these data, and about the significant impact that effective treatment can have on disease progression. The role of managing physicians will be key in providing such education. Currently, physicians may tend to focus more on the long-term efficacy of treatments rather than on patients’ concerns over side effects; however, it is important that physicians managing Asian American patients with CHB are sensitive to the high level of concern over side effects, and are able to provide a balanced explanation of the relative risks of disease progression over those of potential adverse events.

The long-term risk of kidney damage had a large impact on treatment choice. Nephrotoxicity has been reported as a potential concern with adefovir and tenofovir (in the latter case specifically for patients receiving tenofovir for HIV infection).^16^ So far, the effects of these agents on renal function in patients with HBV infection have proved to be mild.^26^ Nevertheless, prescribing information for tenofovir recommends regular monitoring of creatinine clearance in all patients,^16^ and this may present a barrier to uptake among patients concerned about side effects.

Out-of-pocket cost also carried considerable weight. Concerns about the cost of treatment have previously been cited as barriers to HBV screening among Asian American populations.^9^ In this study, Treated participants were more price sensitive than Treatment-naïve participants, probably due to their exposure to the regular monthly outlay on CHB treatment. Results of the cost-sensitivity exercise suggest that participants may be willing to sustain higher monthly out-of-pocket costs than they currently report paying. The influence of cost may be somewhat underestimated in this study, as the sample included a relatively high proportion of participants with relatively high socioeconomic class who were already being followed in a clinic.

Long-term efficacy, time in market, and number of patients worldwide were of relatively low importance to the participants in this study. Education aimed at achieving a better understanding of the importance of these aspects in the treatment of a chronic disease such as CHB may help to overcome the barriers presented by concerns over side effects. There is a growing body of evidence, from clinical and real-world studies, demonstrating that long-term treatment with entecavir or tenofovir results in high rates of virologic response, with minimal resistance rates, reversal of liver disease, and favorable safety profiles.^27^^,^ 28

As with any study of this type, some limitations need to be taken into consideration. The relative importance assigned to the treatment attributes is clearly dependent on the attributes and levels chosen for use in the model. Thus, the risk of kidney disease may have been attributed a particularly high relative importance, because the risk of bone thinning was the only other potential adverse event included. Information about the participants’ current level of liver dysfunction and other comorbidities such as kidney dysfunction was not recorded. This could have impacted participants’ knowledge and choice of answers. However, since possible comorbidities were neither selected for nor against, participants with kidney dysfunction were likely to be in a minority. Sourcing of patients from in-person methods and through multiple sources may have introduced some bias, although the participant population did represent a good balance of gender and socio-economic status. Although levels of higher education and employment were higher in the participant population, this remains representative of the Asian American population studied, since this population has previously been shown to have a higher level of education and employment compared with the overall US population.^29^ Exclusion of patients unwilling to take Western medicines may also have impacted on results; for example, these individuals may have been less knowledgeable about available treatments and/or more concerned about side effects than the participants included in the study. While the specific Asian populations included in the study were chosen to represent those with the highest prevalence of CHB, the inclusion of only Chinese, Korean, and Vietnamese participants may limit the wider applicability of the results to the Asian American population as a whole. However, the lack of differences seen between these populations, in terms of the results of the discrete choice model, suggests that there may be little variation between different ethnicities of the overall Asian American population.

In 2011, the US Department of Health and Human Services published an Action Plan for the Prevention, Care and Treatment of Viral Hepatitis, detailing a comprehensive strategic plan for viral hepatitis prevention and control.^30^ Two key goals were to improve viral hepatitis care and treatment in primary care settings (recognizing that collaborations of primary-care providers and specialists result in the best care for infected persons), and to decrease health disparities by educating communities about the benefits of viral hepatitis prevention and treatment. The results of our study suggest that while most Asian Americans with CHB are aware of the availability of effective antiviral treatment, a considerable proportion (30 %) are not. Even with a high level of awareness of available treatments, Asian American individuals with CHB were reluctant to be treated because of concerns about side effects. It is important that primary care physicians working in Asian American communities take this into consideration when discussing possible treatments with their patients. Targeted education is needed to emphasize the long-term safety and efficacy of available antiviral therapies, highlighting the relatively low risk of serious side effects compared with the life-threatening long-term risks associated with progression of untreated CHB. The role of the treating physician will be essential in providing appropriate counseling and education about CHB and the potential benefits of treatment.

## Electronic supplementary material

Below is the link to the electronic supplementary material.

ESM Fig. 1 Discrete choice model, eight-block, eight-scenario design. Each participant was shown one of 8 blocks. Each block contained 8 different product comparison scenarios. Each block was seen by an equal number of Treated and Treatment-naïve participants. (JPEG 2.24 MB)

High resolution image file (EPS 612 KB)
