# Supplementary figures and images for: Disease and Treatment Perceptions Among Asian Americans Diagnosed with Chronic Hepatitis B Infection
Source: J Gen Intern Med. 2013 Dec 19;29(3):477–84. doi: 10.1007/s11606-013-2673-0 (PMC3930796; doi:10.1007/s11606-013-2673-0)

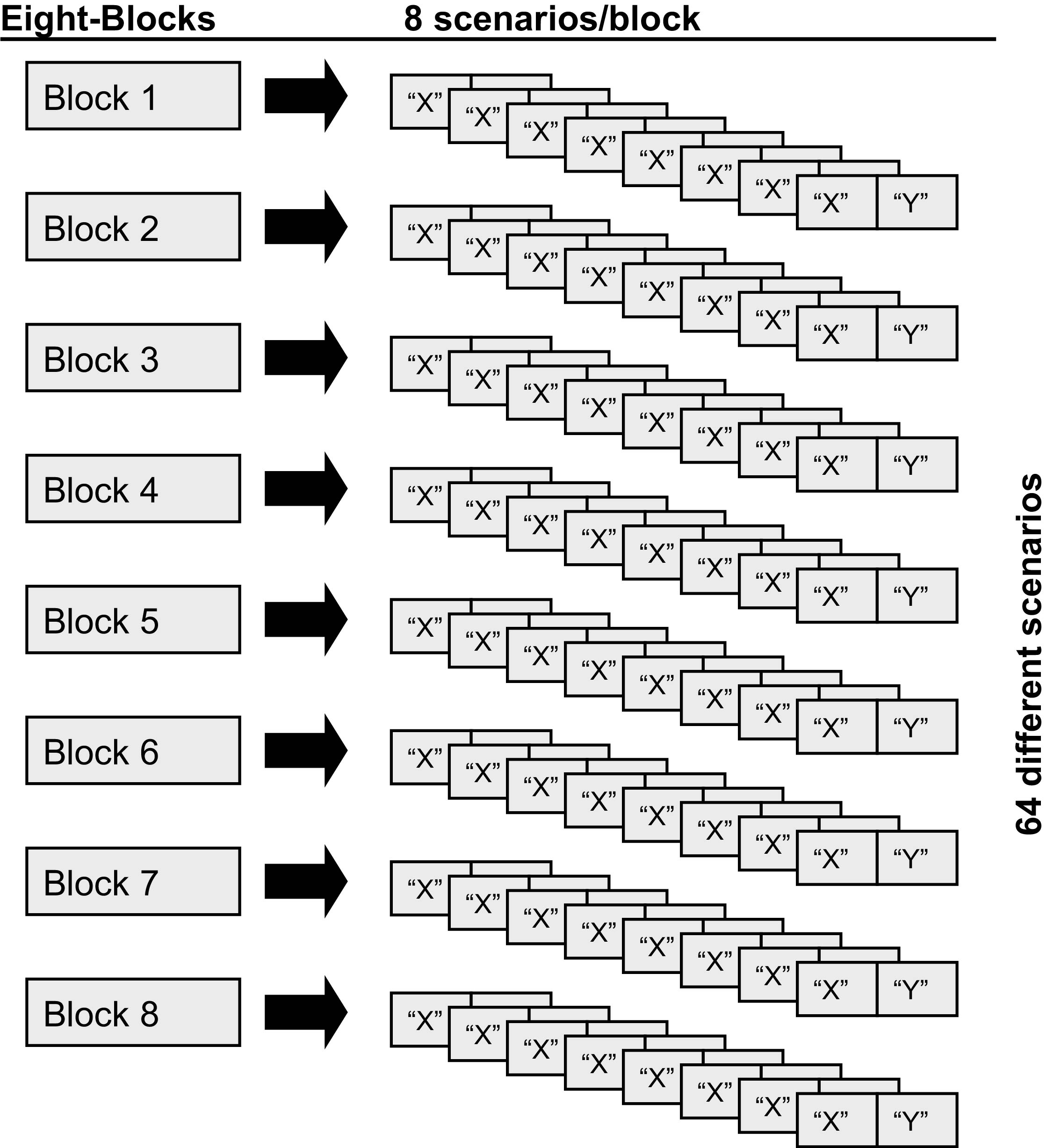

Supplement: Supplementary file 1 — Discrete choice model, eight-block, eight-scenario design. Each participant was shown one of 8 blocks. Each block contained 8 different product comparison scenarios. Each block was seen by an equal number of Treated and Treatment-naïve participants. (JPEG 2.24 MB) [file 11606_2013_2673_Fig4_ESM.jpg]
